# Pathophysiological Association Between Diabetes Mellitus and Alzheimer's Disease

**DOI:** 10.7759/cureus.29120

**Published:** 2022-09-13

**Authors:** Manasi Agrawal, Anil K Agrawal

**Affiliations:** 1 Department of Pathology, Jawaharlal Nehru Medical College, Datta Meghe Institute of Medical Sciences, Wardha, IND

**Keywords:** alzheimer's disease, diabetes mellitus, hypoglycemia, elderly, dementia

## Abstract

Worldwide elderly people are being affected by diabetes mellitus (DM) and dementia. The risk for the development of dementia is higher in people with DM. DM causes a marked cognitive reduction and increases the risk of dementia, most commonly vascular dementia and Alzheimer's disease. People affected by DM and dementia seem to be at higher risk for intense hypoglycemia. Hypoglycemia, the complication of DM treatment, is believed as an independent risk factor for dementia in people with DM. Both Alzheimer's disease and DM are linked with decreased insulin secretion, reduced uptake of glucose, raised oxidative stress, angiopathy, activation of the apoptotic pathway, aging, abnormal peroxidation of lipids, increased production of advanced glycation end products and tau phosphorylation, brain atrophy, and decreased fat metabolism. In this paper, we will review the association between Alzheimer's disease and DM. In addition, we will discuss the agents that enhance the risk for dementia in elderly people with DM and how to prevent the development of cognitive dysfunction in DM.

## Introduction and background

Diabetes mellitus (DM) is a disease in which the body does not generate an adequate amount of insulin or develops insulin resistance, resulting in increased blood sugar levels. In DM, 90% of cases are of type 2 and the remaining are of type 1. It is a consequence of composite interactions among environmental, genetic, and demographic factors. It is generally observed in overweight, middle-aged people. It is characterized by progressively worsening hyperglycemia accompanied by abnormalities of carbohydrate, protein, and fat metabolism. The pathological mechanism responsible for the development of DM is a combination of resistance to the action of insulin in the peripheral target tissues and decreased insulin secretion by beta cells of the pancreas. In the past few decades, there has been a rapid growth of type 2 DM conditions due to high-caloric intake and a decrease in physical activities in the Western world. In the past 30 years, the number of patients with DM has increased six times all over the world, reaching 349 million, and is projected to double over the next 15 years if some preventive measures are not taken [1]. Dementia is a syndrome characterized by decreased level of cognition. Patients with dementia have problems with a variety of cognitive capabilities, most frequently with memory as well as with attentiveness, planning, judgment, attitude, and language [2]. Nowadays, dementia and DM are significant health issues. DM leads to a remarkable cognitive decline and increases the risk of dementia, mainly vascular dementia and Alzheimer's disease, by 100%-150% and 50%-100%, respectively. Amyloid beta (Abeta) is the main pathogenic factor in Alzheimer's disease development. It is eliminated by advanced glycation end products and deteriorated by an enzyme, called an insulin-degrading enzyme, for which it competes with insulin. Insulin activates the secretion of Abeta and advances brain inflammation [3].

Hyperglycemia changes synapse plasticity and leads to cognitive reduction. Advanced glycation end products disturb the function of neurons, and bonding to Abeta elevates its aggregability. Glycation of tau protein stimulates the production of neurofibrillary tangles, which is a primary intracellular pathogenic agent in Alzheimer's disease. Advanced glycation end product 2 in DM causes apoptosis of neurons and angiogenesis. An advanced glycation end-product receptor is a specific Abeta receptor. It produces reactive oxygen species. These reactive oxygen species lead to reduced mitochondrial function and deplete neuronal energy resources. Insulin resistance is linked with a dysexecutive syndrome (a dysregulation of executive functions and is strictly associated with frontal lobe damage), and hyperinsulinemia raises the risk for Alzheimer's disease, primarily by increasing phosphorylation of tau protein and generation of neurofibrillary tangles. Insulin therapy shows improvement in behavior and memory, especially in patients with Alzheimer's disease. Good glycoregulation appears as an essential factor in the prevention of dementia. A better understanding of the relationship between DM and brain functioning will lead to new potential dementia therapies [3].

**Figure 1:**
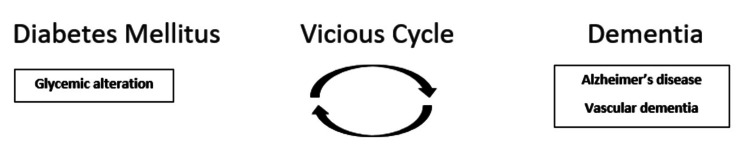
Vicious cycle between diabetes mellitus and dementia. Image credit: Author Manasi Agrawal

How are these two chronic and debilitating disorders indistinguishable? Do they have common denominators? Alzheimer's disease is related to DM in numerous ways. Both are linked with decreased insulin secretion, amyloidosis, reduced uptake of glucose, raised oxidative stress, angiopathy, activation of the apoptotic pathway, aging, abnormal peroxidation of lipids, increased production of advanced glycation end products and tau phosphorylation, brain atrophy, diminished fat metabolism, and pathology of mitochondria [4]. The pathogenesis of both DM and Alzheimer's disease has genetic and environmental constituents. Both can also give rise to dementia. All of these common denominators represent that DM and Alzheimer’s disease share many things in terms of histopathology, pathophysiology, and clinical outcomes. These similarities can be utilized in the search for and formulation of potent pharmacotherapeutic drugs for Alzheimer’s disease, as powerful therapeutic agents such as insulin, oral hypoglycemic agents, incretins, and antioxidants utilized to control DM may play an important role in the management of patients with Alzheimer's disease [4]. DM promotes neurodegeneration by causing alterations in insulin signaling, metabolism of glucose, and vascular functioning and structure. It also alters metabolisms of tau/beta-amyloid. In turn, Alzheimer's disease affects systemic glucose metabolism by influencing alterations in behavior, frailty, memory disturbances, altered functions of the hypothalamus, and possibly alterations in plasma/peripheral Abeta levels. One of the tough challenges experienced during management of people with DM is hypoglycemia, which can further cause neurodegeneration. Due to this vicious cycle, DM and Alzheimer’s disease may lead to neurodegeneration. Figure 1 shows the association between DM and Alzheimer's disease. Different cellular, molecular, interorgan, clinical, and physical agents might contribute to the bidirectional relationship between DM and Alzheimer's disease [5].

Alzheimer’s disease is a neurodegenerative disorder affecting approximately 3.2 crore individuals worldwide [6]. In less than 1% of cases, autosomal-dominant inherited mutations lead to Alzheimer's disease, and these mutations generally lead to the clinical disorder before the age of 61 years. Maximum cases have late-onset Alzheimer's disease, and factors such as genetics, age, environment, and other disorders play an essential role [6]. Alzheimer's disease is marked by a sequence of pathological events involving the production of amyloid plaques, neurofibrillary tangles, brain hypometabolism, synapse loss, neuroinflammation, and atrophy of the brain, which is accompanied by critical and gradual cognitive dysfunction. In the extracellular space, amyloid plaques made up of a cluster of Abeta are produced in a concentration-dependent fashion. The development of hyperphosphorylated and accumulated tau protein accelerates the generation of intracellular neurofibrillary tangles. Aggregation of Abeta appears almost 16 years before a person manifests cognitive reduction. In contrast, the disorder becomes complex before the aggregation of tau protein in the neocortex before the beginning of dementia. Numerous risk factors, genetic as well as nongenetic, for late-onset Alzheimer's disease have been recognized. Genetic changes in the apolipoprotein E (*APOE*) gene have proven to be a potent risk factor for late-onset Alzheimer's disease, apart from aging. The *APOE4* allele enhances Alzheimer’s disease risk by 12 times (two copies) or 4 times (one copy) in part by modifying Abeta aggregation [7]. However, about 50%-60% of patients with Alzheimer's disease have the *APOE4* gene, indicating that more factors are involved in the pathogenesis of Alzheimer's disease [7].

Type 2 DM is the risk factor for late-onset Alzheimer's disease, and the risk of the latter is increased twice [8]. Also a disorder of aging, type 2 DM is identified by hyperinsulinemia, high glucose levels, and insulin resistance. Generally, the binding of insulin to its receptor phosphorylates the insulin receptor substrate on a tyrosine residue. This leads to stimulation of the canonical signaling cascade. In peripheral tissues such as fat, muscle, and liver, this activation finally accelerates the uptake and sequestration of glucose to suffice cellular energy necessities and plays an essential role in the metabolism of lipids [9]. Contradictory to the periphery, where uptake of glucose is mainly dependent on insulin, the brain utilizes nearly 21% of all glucose in the body, i.e., mainly not dependent on insulin [10]. However, brain insulin signaling is strong and has pleiotropic consequences due to insulin receptors, which are predominantly distributed across the brain, and complexity of insulin receptor signaling. For example, regulation of memory can occur by hippocampal stimulation of insulin receptor signaling [10], and insulin receptor signaling in the hypothalamus can alter eating habits and metabolism in the periphery [11]. Similar to Alzheimer’s disease, pathological alterations in insulin occur years before individuals get to know about type 2 DM, which generally occurs once pancreatic beta cell malfunction and resistance to insulin develop chronic high blood sugar levels [12]. Type 2 DM singly has been related to cognitive reduction [13], brain hypometabolism [14], and regional atrophy of the brain [15]. The cognitive decline in type 2 DM is supposed to be mediated by alterations in brain insulin signaling [16]. However, we have less information about people with type 2 DM by measuring insulin/insulin signaling in the central nervous system to assist this affirmation [17]. Type 2 DM can influence the risk of Alzheimer's disease in the following ways: (1) type 2 DM can give rise to small blood vessel disorders, which can cause dementia, independent of or along with Alzheimer's disease pathology, by disturbing the appropriate functioning of the vasculature of the brain [18], and (2) type 2 DM produces alterations in the brain function directly or interact with the essential proteins or pathways associated with the pathology of Alzheimer's disease, such as tau or Abeta. Over the past 16 years, many researchers have proposed alterations in insulin levels or insulin signaling in people with late-onset Alzheimer's disease, recommending that people with Alzheimer's disease experience increased insulin levels and resistance to insulin in the brain. From the aforementioned discussion, it can be interpreted that resistance to insulin in the brain develops due to Alzheimer's disease pathology. High insulin levels are a compensatory mechanism leading to type 3 DM [19]. 

## Review

Population

Worldwide, individuals are being affected by dementia and DM [20]. Recent research conducted in Canada reveals that approximately 21% of the elderly Canadians are affected by DM, and as DM is frequently asymptomatic in these citizens, the definite number is probably elevated. At least 16% of elderly individuals have dementia, and this number is predicted to rise in the succeeding decade. The spread of mild cognitive dysfunction, a precursor to dementia, is also increasing. Many investigations reveal that the risk of dementia is doubled in individuals with DMs [21]. This is correct for both vascular and Alzheimer's dementia. Other contributing agents include hypertension, microvascular and macrovascular disorders, resistance to insulin, chronic inflammation, hyperglycemia, frequent severe hypoglycemia, increased levels of cortisol, and genetic predisposition [20].

The spread of DM appears to be increasing continuously because of inappropriate diets and sedentary lifestyles. DM is the most common metabolic disorder affecting >250 million individuals worldwide [4]. It is predicted that the number of individuals affected by DM will continue to rise in the coming decades. Alzheimer's disease is the leading cause of dementia and influences >25 million individuals globally, generally the elderly. The worldwide spread of Alzheimer's disease is believed to double in the next 21 years [4]. Epidemiological studies have pointed out an association between DM and risk of developing dementia. A separate population-based prospective group study has been conducted on elderly inhabitants of the town of Hisayama in Japan since 1986, pointing out secular trends in the spread of dementia and inspecting the risk and protective agents for dementia in the Japanese population. In a prospective study of risk factors for dementia in elderly citizens without dementia in Hisayama, DM was recognized as an essential risk factor for generation of the all-cause dementia, especially Alzheimer's disease. In addition, two-hour post-load sugar levels were firmly related to an elevated risk of all-cause dementia, vascular dementia, and Alzheimer's disease. In a pathological study of Hisayama inhabitants, increased levels of two-hour post-load sugar, fasting insulin, and homeostasis model assessment of insulin resistance were remarkably related to enhanced risk of neuritic plaques. The steep elevation in the frequency of DM could lead to the increasing trend in the spread of dementia, especially Alzheimer's disease, in older Japanese people [22].

Risk Factors

**Figure 2:**
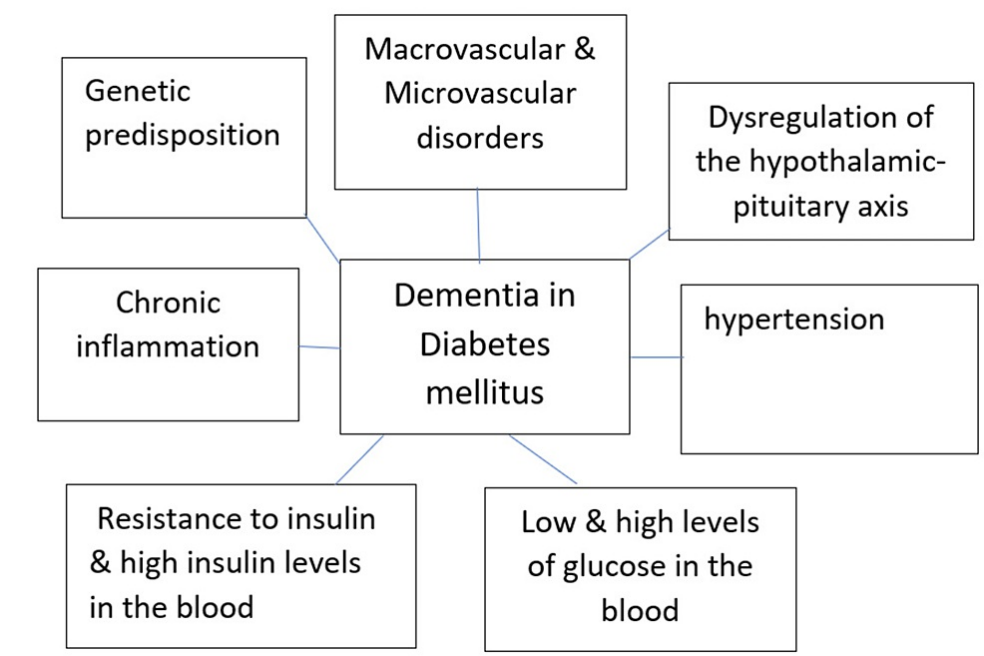
Pathophysiologic factors related to dementia and diabetes mellitus in the elderly. Image credit: Author Manasi Agrawal

The most prevalent types of dementia are not only Alzheimer's disease but also vascular dementia, and a majority of people are affected by a mixed type of dementia. It makes appropriate sense to suppose that DM increases the risk for a vascular or mixed type of dementia, given its effect on the vascular system, and occurrence of vascular dementia is surely enhanced in people with type 2 DM. Anyway, the widespread of Alzheimer's disease may also increase in people with DM; however, the odds ratios are fewer than those for vascular dementia [23]. Various agents may give rise to dementia in people with DM. Figure 2 shows the pathophysiologic factors related to dementia and DM in the elderly. First, people with DM are recognized to be at high risk for stroke and small blood vessel disorders, which can give rise to mixed or vascular dementia. In favor of this observation, it is noted that the appearance of macrovascular or microvascular problems and hypertension raises the risk of dementia [24]. Chronic hyperglycemia, as indicated by increased amounts of glycated hemoglobin, is linked with impaired cognition in people with DM [25]. Probably, oxidative stress, microvascular changes, changes in synaptic plasticity, and aggregation of advanced glycation end products are promoting agents to these cognitive consequences. Interestingly, there seems to be a potent association between risk of dementia and amounts of postprandial hyperglycemia. Contrarily, severe hypoglycemia may also elevate the risk of dementia.

There is a correlation between hyperinsulinemia, resistance to insulin, and cognitive dysfunction. Insulin has neurotropic properties. It is swiftly transferred beyond the blood-brain barrier. Receptors of insulin are concentrated primarily on different areas of the brain that are related to memory and learning. Insulin is required to generate some significant neurotransmitters, and nasal insulin has appeared to enhance memory in people with cognitive dysfunction [26].

How Does Resistance to Insulin and Hyperinsulinemia Cause Loss of Memory?

Insulin resistance results in downregulation of receptors of insulin, which causes the neurotropic effect of insulin on the brain to decrease. Insulin resistance is related to a high amount of cytokines, decreased uptake of sugar, and decreased flow of blood. Moreover, hyperinsulinemia may change amyloid metabolism, leading to its aggregation and deleterious effect. Chronic inflammation is present in most people with DM, and the increased amount of cytokines is related to poorer cognition in people with DM [27]. It is possible that high amounts of inflammatory cytokines give rise to dementia by having a direct impact on the brain, by giving rise to a vascular disorder, or by causing resistance to insulin, with the aforementioned consequences [28]. People with type 2 DM have dysregulation of the hypothalamic-pituitary axis and increase amounts of cortisol. There is a correlation between elevated amounts of cortisol and cognitive impairment in people with type 2 DM. Elevated amounts of cortisol are linked with microvascular deformities in people with DM. Along with the effect on circulation, increased amounts of cortisol may have harmful consequences on the hippocampus. Alterations in the brain and depletion of cognitive scores are most recognizable in people with DM who possess the *APOE4* allele, providing additional correlation between dementia and DM and suggesting that genetic constituents can play an essential role in this relation [29]. Rheologic deformities may also play an important role. Elevated amounts of rheologic factors can elevate the resistance to flow and are linked with vascular diseases. For example, there are a few proofs of correlation between raised viscosity of plasma and diminished cognition in people with type 2 DM. Even if Alzheimer's disease and type 2 DM are two independent disorders, proofs from pathophysiological, epidemiological, and animal studies have suggested a strong pathophysiological association between these disorders. Due to the pathophysiological resemblance of Alzheimer's disease and type 2 DM, which involves resistance to insulin and insufficiency of insulin, protein accumulation, inflammation, advanced glycation end products, oxidative stress, and autophagocytosis, Alzheimer's disease is frequently mentioned as a "type 3 DM" [30].

In addition to enhanced risks of microvascular and macrovascular problems, individuals with type 2 DM are also at enhanced risks of dementia and cognitive dysfunction. Hypoglycemia, a complication of DM therapy, is observed as a not dependent threat factor for dementia in people with type 2 DM. Dementia and hypoglycemia are clinically underestimated and are associated with poor consequences; hence, they may compromise the life expectancy of individuals with type 2 DM. Epidemiological proof of hypoglycemia-related cognitive reduction and dementia is highly diverse. Acute, severe hypoglycemic attacks produce chronic subclinical brain impairment, cognitive reduction, and subsequent dementia. Although the consequences of repeated moderate hypoglycemia on cognitive reduction and dementia persist, they remain primarily uninvestigated. Poor glycemic management (including alteration of hemoglobin A1C and glucose values) and the vicious cycle of a bidirectional relation between hypoglycemia and dementia may be clinically appropriate. The possible pathophysiological proposition involves posthypoglycemic neuronal injury, coagulation abnormality, endothelial deformities, inflammatory processes, and synaptic dysfunction of hippocampal neurons during hypoglycemic attacks. This report discusses earlier findings, provides insights into the recognition of groups at increased risks of hypoglycemia-related dementia, and suggests specific strategies to decrease potential load linked with hypoglycemia-associated neurocognitive disorders in people with type 2 DM [31].

Prevention

How Is the Development of Cognitive Impairment in DM Prevented?

Better glycemic management would appear to be an excellent idea, but ACCORD-MIND found that better control resulted in no alterations in cognition [32]. Prevention of depletion in blood glucose levels would seem to be cautious, but currently, there are no publicized reviews to promote this presumption. However, it would be logical that the management of various risk factors should decrease dementia risk; the utilization of antiplatelet agents and statins does not result in the depletion in the risk of dementia in the elderly without DM. The Hypertension in the Very Elderly Trial cognitive function assessment (HYVET-COG) study recommends that hypertension management possibly has a positive effect on cognition in the elderly without DM [33], and there are a few proofs that management of hypertension can inhibit the cognitive reduction in people with DM. Work on the animal has recommended that certain drug types used in DM, especially glucagon-like peptide-1 (GLP-1) analogs and glitazones, might have a favorable impact on cognition. Still no up-to-date good human data are available to recommend positive results from any specific drug classes. Nasal insulin enhances cognitive performance in people with dementia, but this has not been observed in older adults with DM. As per preliminary data, depletion in the amount of cortisol possibly has a good effect on cognition in people with DM. In preventing cognitive impairments in DM, an improved solution needs to be found by us. As exercise inhibits both DM and dementia in the elderly [34], it is completely believable that exercise will decrease the threat of cognitive dysfunction in people with DM. However, exercise does not seem to enhance the cognitive function in people with dementia who do not have DM; there is some preliminary proof that exercise may suppress aspects of cognitive reduction in the elderly with irregular metabolism of glucose, which are not yet demented [35].

In accession to targeted regimens utilized usually for treatment of Alzheimer's disease and type 2 DM individually, at present, drugs for diabetes are effectively utilized to decrease cognitive reduction in people with Alzheimer's disease. Hence, if the ordinary pathophysiology of Alzheimer's disease and type 2 DM could be clearly determined, the two disorders could be controlled further effectively, hopefully by shared pharmacotherapy in addition to understanding a broader spectrum of preventative strategies. The purpose of this paper is to study the pathophysiological link between type 2 DM and Alzheimer's disease to lay the foundation for upcoming treatment strategies in the regulation of both disorders [30].

## Conclusions

The risk of dementia doubles in people with DM. Several factors enhance the risk of dementia in people with DM such as microvascular and macrovascular diseases and hypertension. Alzheimer's disease is related to DM in numerous ways, in that both are linked with decreased insulin secretion, amyloidosis, reduced uptake of glucose, raised oxidative stress, angiopathy, activation of the apoptotic pathway, aging, abnormal peroxidation of lipids, increased production of advanced glycation end products and tau phosphorylation, brain atrophy, diminished fat metabolism, and mitochondrial pathology.
